# Perampanel‐associated exacerbation of de novo psychosis and lateralized rhythmic delta activity: A case report

**DOI:** 10.1002/pcn5.70208

**Published:** 2025-09-23

**Authors:** Yu Fujiwara, Tomohiro Iwata, Takero Terayama, Shogo Takeshita, Aihide Yoshino

**Affiliations:** ^1^ Department of Psychiatry School of Medicine, National Defense Medical College Tokorozawa Saitama Japan; ^2^ Department of Emergency Medicine Self Defence Forces Central Hospital Setagaya‐ku Tokyo Japan

**Keywords:** encephalopathy, lateralized rhythmic delta activity, perampanel

## Abstract

**Background:**

Perampanel (PER) may instigate psychiatric phenomena encompassing irritability and aggression. This study elucidates an epileptic patient in whom PER appeared to exacerbate preexisting psychosis, concomitant with lateralized rhythmic delta activity (LRDA).

**Case Presentation:**

A 30‐year‐old right‐handed female initially exhibited focal aware seizures and focal to bilateral tonic–clonic seizures at 21 years of age. Despite administration of multiple antiseizure medications (ASM), she continued to experience weekly seizure episodes. At the age of 26 years, she underwent right selective amygdalohippocampectomy (SeAH) concurrent with vagus nerve stimulation (VNS) implantation. Following this intervention, the seizure frequency diminished from weekly to monthly, albeit not entirely eliminated. Five months postoperatively, the patient displayed paranoid delusions and auditory hallucinations. Her psychotic symptoms were assessed as de novo psychosis. In July of year Y‐1, PER was coadministered with Valproic acid, Lacosamide, and Levetiracetam to optimize seizure control, with the dosage escalated to 6 mg by December of year Y‐1. Simultaneously, in July of year Y, heightened irritability, aggression, and psychomotor agitation became prominent, necessitating hospitalization in August of year Y. An electroencephalogram (EEG) upon admission revealed LRDA over the right posterior quadrant. Subsequent cessation of PER administration culminated in the resolution of both exacerbated psychiatric symptoms and LRDA within approximately a week.

**Conclusion:**

The observed LRDA in this case may represent cerebral dysregulation, possibly induced by PER, concurrent with the worsening of psychiatric sequelae. Psychosis during PER treatment could signify underlying brain dysfunction, highlighting the potential utility of EEG monitoring in managing these patients.

## Graphical abstract

This case study describes a 30‐year‐old female with refractory focal seizures who developed psychotic symptoms, irritability, and aggression after the initiation of perampanel (PER). Notably, these symptoms and the associated lateralized rhythmic delta activity (LRDA) resolved upon PER discontinuation, suggesting a direct link between PER‐induced brain dysfunction and psychiatric manifestations. These findings highlight the importance of monitoring LRDA and psychiatric symptoms in PER patients.

## BACKGROUND

The antiseizure medication perampanel (PER) has been implicated in the emergence of psychiatric manifestations, notably irritability, aggression, and depressive symptoms. However, the underlying pathophysiological mechanisms remain incompletely understood.^1^ This study elucidates a case in which PER appeared to exacerbate a preexisting neuropsychiatric disorder, accompanied by lateralized rhythmic delta activity (LRDA).

## CASE PRESENTATION

A 30‐year‐old right‐handed female reported feeling harassed during her initial assessment. Her family medical history was unremarkable, but she had meningitis 10 years previously. Her development was normal, but her childhood was marred by the physical abuse of her father. After finishing high school, she worked as a cashier. However, after epilepsy surgery, frequent seizures and emotional instability hindered her ability to maintain a job, leading her to join a day care program. She was previously described as bubbly and friendly.

**Figure 1:**
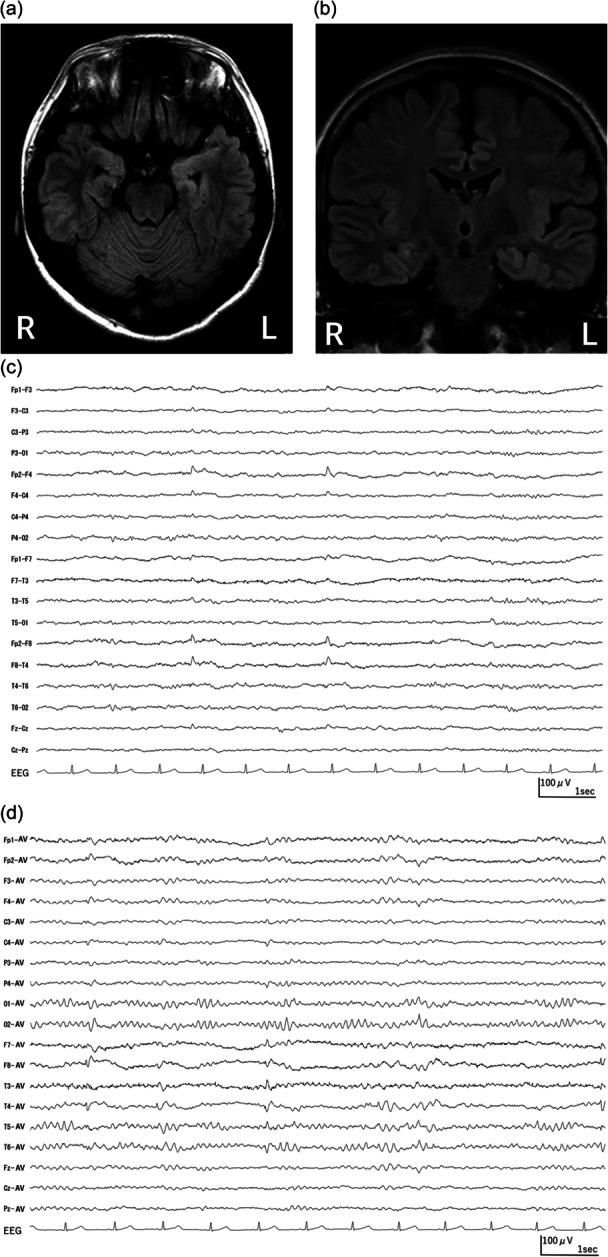
Presurgical brain magnetic resonance imaging (MRI) and electroencephalography (EEG) findings. (a) Axial FLAIR (fluid‐attenuated inversion recovery) brain MRI revealing bilateral mild hyperintensity in the hippocampi. No other abnormalities, including focal cortical dysplasia, were identified. (b) Coronal FLAIR brain MRI demonstrating bilateral mild hippocampal hyperintensity and blurring of the internal hippocampal lamination, suggestive of hippocampal sclerosis. No other abnormalities, including focal cortical dysplasia, were observed. (c) EEG recorded using a longitudinal bipolar montage (sensitivity: 10 µV/mm, high‐cut filter: 120 Hz, time constant: 0.3 s). Repetitive sharp waves were observed predominantly in the right anterior temporal region, particularly during drowsiness. (d) EEG recorded using an average reference montage (sensitivity: 10 µV/mm, high‐cut filter: 120 Hz, time constant: 0.3 s). Background activity exhibited a symmetrical 10 Hz alpha rhythm, predominantly in the occipital region. Sharp waves were also noted in the left anterior temporal region.

In May of year Y‐9 (nine years before the current presentation), the patient was admitted to the Internal Medicine Department with symptoms of tonic–clonic seizures. The patient presented with paroxysmal episodes characterized by an ascending epigastric sensation, palpitations, and a feeling of fear. Occasionally, these episodes progressed to impaired awareness with a fixed stare. Infrequently, they evolved into focal to bilateral tonic–clonic seizures, which were heralded by conjugate eye deviation to the left, followed by tonic posturing involving extension of the left upper limb, flexion of the right upper limb, and extension of both lower limbs. This tonic phase was succeeded by bilateral clonic seizures. The electroencephalogram (EEG) revealed sharp‐wave discharges predominantly in the right temporal region, with fewer also noted in the left temporal region. Brain magnetic resonance imaging (MRI) demonstrated findings consistent with bilateral hippocampal sclerosis (Figure 1). Based on these findings, a diagnosis of right temporal lobe epilepsy was established. The patient experienced seizures on a weekly basis. A comprehensive treatment plan, including levetiracetam (1000 mg), carbamazepine (200 mg), clonazepam (1 mg), valproic acid (1000 mg), gabapentin (2400 mg), and their combined use, was implemented. However, this plan was ineffective in controlling seizures, and the seizure frequency remained weekly.

On March Y‐5, following a diagnosis of drug‐resistant epilepsy, the patient was transferred to the Neurosurgery Department for surgical intervention evaluation.

Epilepsy focus resection was initially not considered indicated due to suspected bilateral seizure onset based on EEG and MRI. A vagus nerve stimulator was implanted. However, seizure frequency remained unchanged on a weekly basis. In the following year, due to the strong desire from the patient and family for a focus resection to reduce seizure frequency, and after confirming the epileptogenicity of the right temporal lobe through electrode placement, right selective amygdalohippocampectomy (SeAH) was performed. As a result, seizure frequency decreased from weekly to monthly. Comprehensive neuropsychological evaluations were conducted before and after surgery using the WAIS‐III and the WMS‐R. The FIQ score was 62 preoperatively and slightly lower at 55 postoperatively, confirming no significant deficits in higher cognitive functions. A revised treatment combination, including lacosamide (300 mg), levetiracetam (3000 mg), valproic acid (800 mg), and acetazolamide (500 mg), was administered postoperatively, but the seizure frequency remained monthly.

In February Y‐3 (approximately 5 months postoperatively), the patient began manifesting paranoid delusions and auditory hallucinations, characterized by beliefs such as “I am being manipulated through VNS,” “My neighbors are going to kill me,” and “Everyone maligns me behind my back.” Psychotic symptoms were not present preoperatively and persisted for more than 1 month, independent of seizures; therefore, it was diagnosed as de novo psychosis. Consequently, in March Y‐3, she was readmitted to the psychiatric unit of Hospital B. In the same month, risperidone 1 mg was prescribed to address her delusional perceptions. As a result, her psychiatric symptoms improved to the extent that she could engage in social life without problems, and she was discharged.

In July Y‐1, in an effort to further suppress epileptic seizures, perampanel was introduced and titrated to a dose of 6 mg by December Y‐1. However, the seizure frequency remained monthly. Around July of year Y, her delusions intensified, particularly those pertaining to potential poisoning and overarching paranoia. She was admitted to the hospital in August Y due to marked irritability, aggression, psychomotor agitation, and refusal to take medication associated with delusions of poisoning.

Upon admission, the patient's clinical presentation was as follows. Despite repeated counseling regarding the imperative of medication adherence, the patient remained resolute in her refusal, displaying an increasingly rigid demeanor. Her agitation escalated, culminating in psychomotor excitement during which she assaulted her father and thrashed her upper extremities. Concomitantly, she exhibited pronounced psychotic symptoms, and the concomitant discontinuation of antiseizure medications exacerbated the frequency of epileptic episodes. Her resistance to treatment was underscored by delusional beliefs that there was a widespread conspiracy to harm her, with sentiments such as “all are complicit in plotting against me” and “they intend to adulterate my medication.” In response to her attempts to abscond in tandem with violent outbursts, the medical team administered an intravenous injection of 2 mg flunitrazepam to induce sedation, facilitating her transfer to an inpatient facility. Throughout her inpatient tenure, she persisted in opposition to medical evaluations, rendering neuropsychological assessments unfeasible. The patient required seclusion and restraint due to overt paranoia, heightened psychomotor activity, and aggressive behavior.

Upon initial assessment, the patient was administered the following oral pharmacological agents: Perampanel (6 mg), Lacosamide (300 mg), Levetiracetam (3000 mg), Valproic acid (800 mg), and Acetazolamide (500 mg). Laboratory evaluations yielded normative results, devoid of any indications of hepatic or renal insufficiency or any augmented inflammatory markers. The serum concentration of valproic acid was quantified at 39.7 ug/mL, confirming that she had been adhering to her oral medication regimen until at least a few days prior to admission.

**Figure 2:**
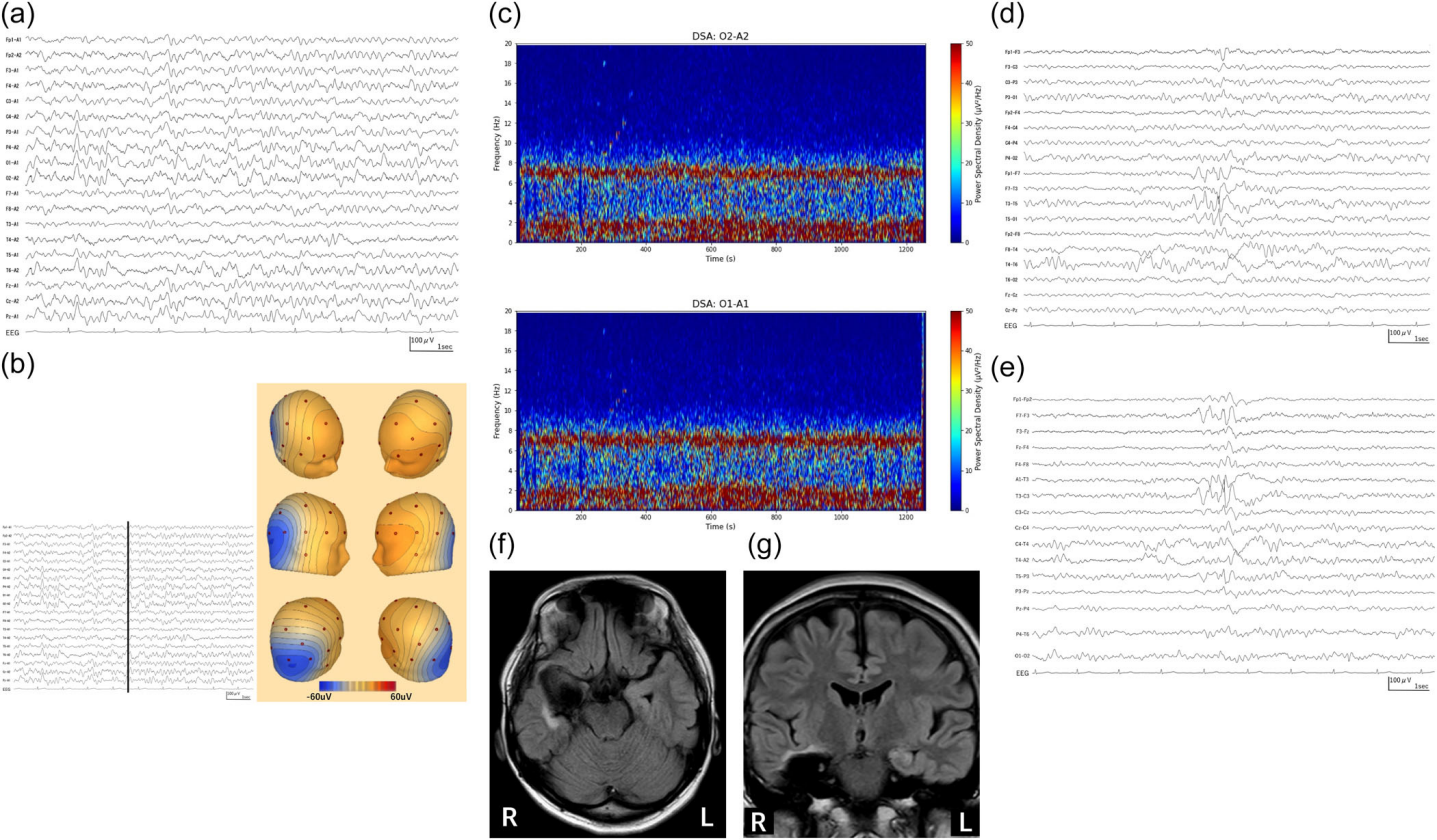
Electroencephalographic (EEG), quantitative analysis demonstrating lateralized rhythmic delta activity (LRDA), and brain magnetic resonance imaging (MRI) findings upon admission. (a) Earlobe referential montage EEG. The recording (sensitivity: 10 µV/mm, high‐cut filter: 120 Hz, time constant: 0.3 s) displays a background of persistent 6–7 Hz theta activity. Superimposed on this is lateralized rhythmic delta activity (LRDA) at approximately 2 Hz, which is maximal in the right posterior temporal region (e.g., O2–A2, T6–A2 channels). The activity was classified as nonictal due to the lack of clinical or electrographic evolution. (b) Scalp potential topography of the LRDA. The compressed EEG trace on the left shows the analyzed segment. The vertical black line indicates the specific time point for the potential maps displayed on the right. The series of six maps illustrates the spatial distribution of the delta wave, with a color scale from blue (−60 µV) to red (+60 µV). These maps consistently demonstrate a negative potential field centered over the right posterior quadrant, providing spatial evidence for the origin of the LRDA. (c) Density spectral array (DSA) of the O2–A2 and O1–A1 channels. This quantitative analysis displays frequency on the *Y*‐axis (0–20 Hz) and time on the *X*‐axis, with signal power color‐coded from blue (0.0 µV²) to red (50.0 µV²). The DSA objectively confirms the sustained and rhythmic nature of the LRDA, revealing a prominent and continuous high‐power band at approximately 2 Hz. This is distinct from the 6–7 Hz background theta activity and contradicts the interpretation of the delta activity as isolated or single waves. (d) EEG with a longitudinal bipolar montage. (e) EEG with a transverse bipolar montage. (sensitivity: 10 µV/mm, high‐cut filter: 120 Hz, time constant: 0.3 s). The EEGs (c) and (d) demonstrate sharp waves. Sharp waves persisted in the left temporal region; this finding was assessed as not representing forced normalization. (f) Axial fluid‐attenuated inversion recovery (FLAIR) brain MRI. (g) Coronal FLAIR brain MRI. The brain MRI at admission (a, b), without contrast administration, revealed findings consistent with a right temporal lobectomy. Preexisting mild hyperintensity on FLAIR images and partial blurring of the hippocampal structure in the left hippocampus, suggestive of hippocampal sclerosis, were noted. No other significant abnormalities were identified.

The EEG and brain MRI were performed (Figure 2). The EEG revealed a background rhythm predominantly characterized by persistent theta waves at 6–7 Hz. Superimposed on this background, notable rhythmic delta activity at approximately 2 Hz was most pronounced in the right posterior temporal regions, indicative of LRDA. Additionally, as sharp waves persisted in the left temporal region, this was assessed as not representing forced normalization. The EEG was evaluated as nonictal, as no evolution was detected. The brain MRI, performed without contrast administration, revealed findings consistent with a previous right temporal lobectomy. Apart from preexisting mild hyperintensity on fluid‐attenuated inversion recovery (FLAIR) images and partial blurring of the hippocampal structure in the left hippocampus, suggestive of hippocampal sclerosis, no additional abnormalities were identified.

**Figure 4:**
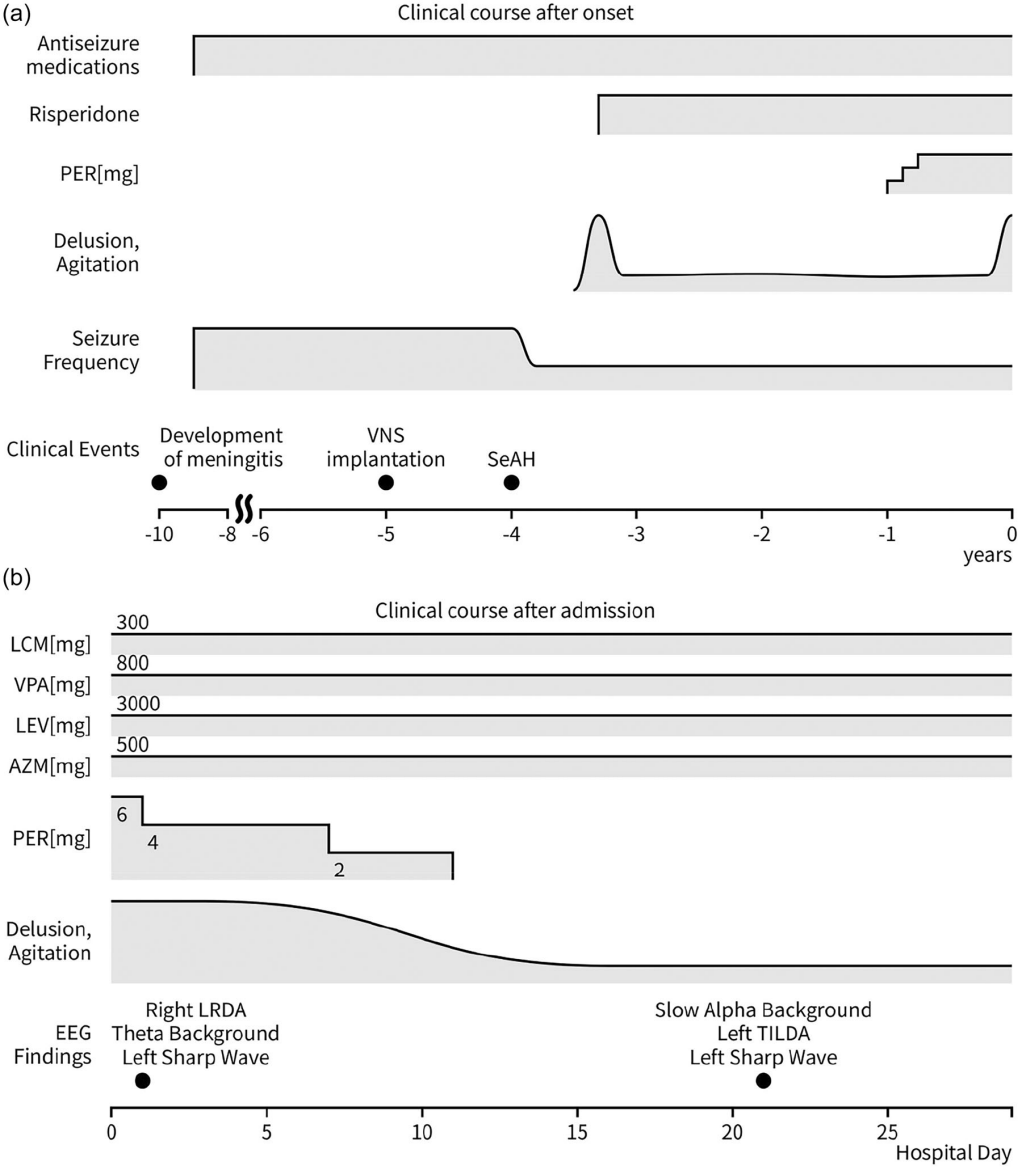
Clinical course of the patient from epilepsy onset through hospitalization to discharge. (a) Clinical course before admission. The patient developed meningitis in year Y‐10. In year Y‐9, she developed focal aware sensory seizures and focal to bilateral tonic–clonic seizures. Despite the administration of multiple antiseizure medications (ASMs), she continued to experience weekly seizure episodes. She underwent vagus nerve stimulation (VNS) implantation in year Y‐5 and right Selective amygdalohippocampectomy (SeAH) in year Y‐4. After SeAH, the seizure frequency diminished from weekly to monthly, albeit not entirely eliminated. Five months postoperatively, the patient displayed paranoid delusions and auditory hallucinations. In year Y‐1, perampanel (PER) was coadministered with valproic acid, lacosamide, and levetiracetam to optimize seizure control, with the PER dosage escalated to 6 mg. Approximately 1 year later, heightened irritability, aggression, and psychomotor agitation became prominent, necessitating hospitalization in August of year Y. (b) Clinical course after admission. An electroencephalogram (EEG) upon admission revealed lateralized rhythmic delta activity (LRDA) over the right posterior quadrant. Subsequent cessation of PER administration resulted in the resolution of both exacerbated psychiatric symptoms and LRDA within approximately 2 weeks. An EEG performed after symptomatic improvement showed amelioration of background slowing, and the right‐sided LRDA was almost absent. Left‐sided sharp waves remained largely unchanged from those observed at admission.

The onset of perampanel coinciding with the intensification of the patient's restlessness suggested possible drug‐induced encephalopathy. The patient had been prescribed 6 mg of perampanel as an outpatient, which was reduced to 4 mg on the day of admission, further reduced to 2 mg on day 7, and discontinued on day 11. By day 7, a noticeable improvement in paranoia and psychomotor agitation was observed, along with consistent medication compliance. As a result, the patient was discharged from isolation on day 8. On the same day, significant improvements in attention and orientation were noted, despite a clear amnestic phase from preadmission to day 7. A follow‐up EEG on day 21 demonstrated significant improvement in right LRDA (Figure 3), suggesting that encephalopathy, likely induced by perampanel, was presumably responsible for the observed hallucinations, delusions, and psychomotor hyperactivity. After discontinuation of perampanel, there was no change in seizure frequency compared to the period preceding hospitalization. Since perampanel did not affect seizure frequency either after its initiation or after its discontinuation, it was assessed as having no effect on epileptic seizures. The patient had no memory of the period from approximately 1 month before hospitalization until the psychiatric symptoms subsided. After confirming the improvement of psychiatric symptoms and the patient's ability to take oral medications without issues, the patient was discharged on day 29 after confirming the absence of psychiatric symptoms (Figure 4).

Figure 3The electroencephalogramic (EEG) findings on day 21 following symptomatic improvement. (a) Earlobe reference montage. (b) Transverse bipolar montage. (EEG parameters: sensitivity 10 µV/mm, high‐cut filter 120 Hz, time constant 0.3 s). This follow‐up EEG on day 21 demonstrated significant improvement in the previously noted right‐sided lateralized rhythmic delta activity (LRDA). The background activity, primarily shown in panel (a), was normalized to an 8–9 Hz slow alpha rhythm predominantly in the occipital region—an improvement compared to the EEG on admission. Furthermore, as depicted in panel (b), left temporal intermittent rhythmic delta activity was observed, which was interpreted as TILDA; this activity consisted of somewhat rhythmic 2–3 Hz delta waves lasting for approximately 10 s and was considered suggestive of epileptogenicity. Sharp waves in the left temporal region persisted. (c) Density spectral array (DSA) of the O2–A2 and O1–A1 channels. In stark contrast to the findings upon admission (Figure 2c), this DSA recorded following perampanel withdrawal demonstrates the disappearance of the high‐power 2 Hz delta band, objectively confirming the resolution of the LRDA. The background rhythm has also consolidated into a dominant band within the 8–9 Hz alpha frequency, indicating the normalization of brain activity.

**Figure:**
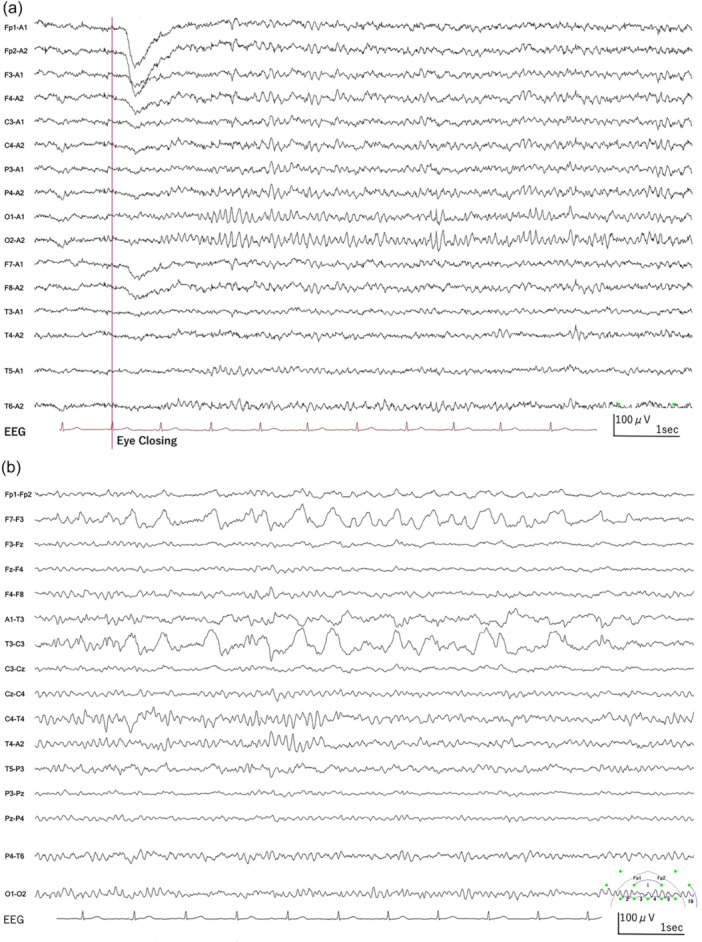

**Figure:**
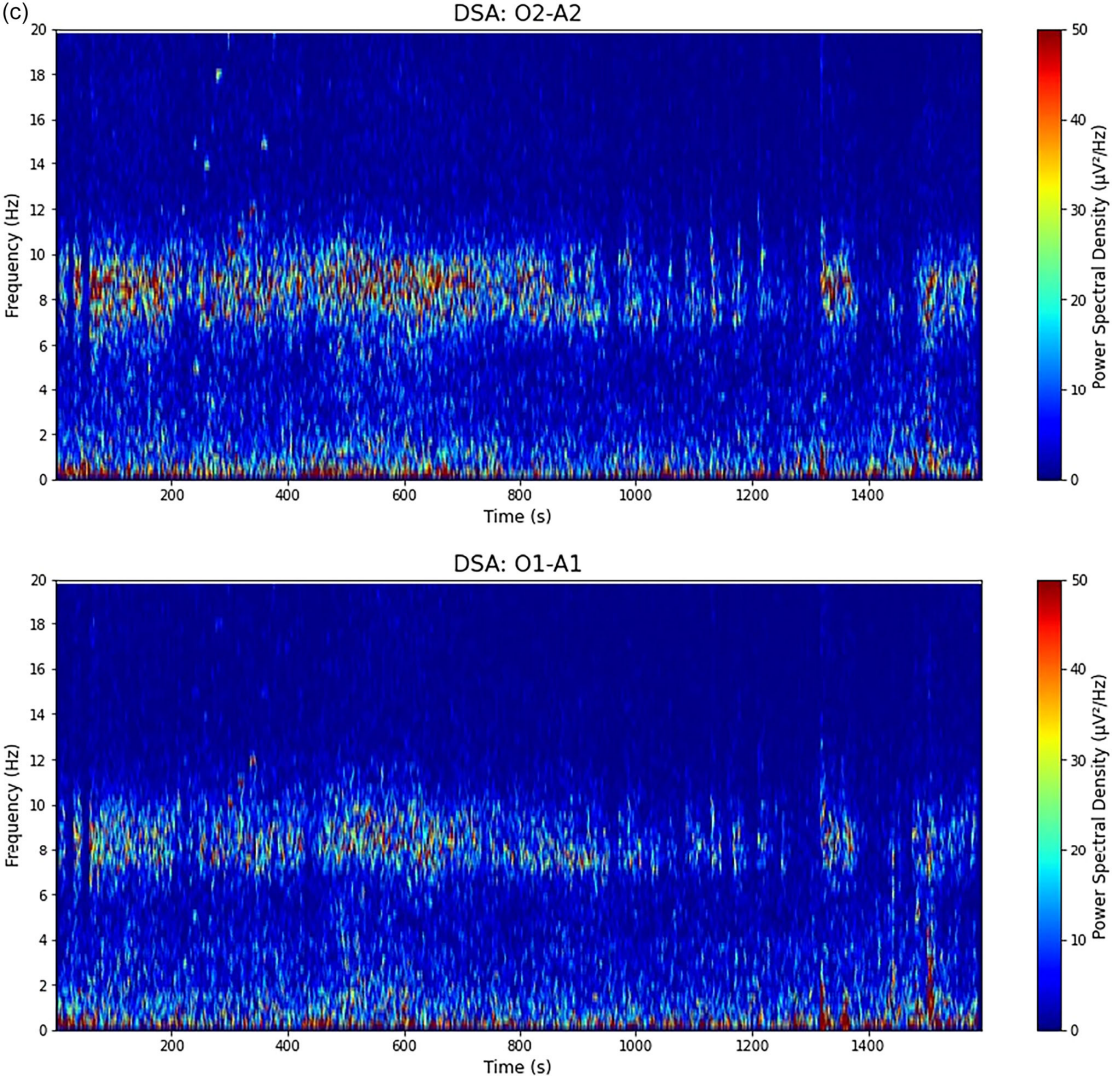

## DISCUSSION

### Psychiatric symptoms related to perampanel

The precise pharmacodynamics underlying the psychiatric symptoms of perampanel remain unclear. However, emergent hypotheses suggest that alternate psychosis is linked with EEG normalization and seizure amelioration, alongside amplified aggression due to inhibited glutamatergic transmission via AMPA receptors.^2^

Unlike levetiracetam (LEV), perampanel (PER) predominantly increases aggression and irritability, excluding manifestations, such as depression, agitation, and cognitive dysfunction.^3^ Approximately 9.2% (range: 1.9%–20.4%) of individuals find sustained PER administration challenging due to increased aggression.^4^ Such psychiatric effects typically manifest within 6 months of initiation and persist for weeks, even after dose modification or discontinuation.^2^, ^5^

The neuropsychiatric effects of PER exhibit interindividual variability, with pronounced prevalence in individuals predisposed to psychiatric disorders, such as the present patient with a history of postoperative psychosis and cognitive deficits.^6^, ^7^ Notably, the impact of PER remained consistent, regardless of the degree of intellectual impairment.^8^

CNS‐related adverse events from PER, although frequently dose‐dependent, have been reported, even at dosages as low as 2–4 mg/day.^9^

In the context of psychiatric symptoms related to antiseizure medications, the concept of alternative psychosis associated with EEG normalization and seizure reduction has also been discussed. This condition typically involves an improvement in seizure control concurrent with the onset of psychotic symptoms.^10^

### EEG changes potentially induced by perampanel

In prior studies, EEG background activity demonstrated deceleration in the theta frequency range, commensurate with serum concentrations of PER.^11^ In contrast, another study elucidated a decrement in the theta band coupled with augmentations in both the alpha and beta frequency ranges 3 months postinitiation of PER.^12^ Notably, no manifestations of LRDA have been documented in these studies.

### About LRDA

In an analysis of four cases marked by LRDA, a bifurcation became discernible: two patients exhibited herpetic encephalitis, while the other exhibited autoimmune encephalitis. Notably, each instance displayed insular cortical aberrations, concomitant with the onset of acute symptomatic seizures.^13^ LRDA has been further portrayed as a quintessential manifestation within the ictal–interictal spectrum of acute symptomatic seizures.^14^ Individuals afflicted with LRDA frequently exhibit focal impairments localized to the hemisphere of rhythmic activity.^15^ Such observations accentuate a salient correlation between LRDA and heightened susceptibility to acute symptomatic seizures and focal impairment.

### Case overview and interpretation

Under familial oversight, the patient adhered to the prescribed regimen without any evidence of overdose or substance dependence. The patient's history of interictal psychosis and moderate cognitive deficits heightened their susceptibility to psychiatric sequelae. Approximately 1 year after the dose of perampanel (PER) reached 6 mg, the patient experienced an acute exacerbation of psychomotor agitation and psychotic symptoms. Although the patient had preexisting postoperative psychotic symptoms, there was a clear deterioration characterized by markedly increased aggression and exacerbated persecutory delusions. Furthermore, these symptoms improved rapidly upon discontinuation of PER, and their exacerbation was temporally correlated with PER treatment. This acute episode could not be attributed solely to the patient's baseline postoperative psychosis or recurrent nonconvulsive seizures and was thus diagnosed as PER‐exacerbated psychosis.

Consistent with these exacerbated psychiatric symptoms, the patient's EEG showed LRDA (Figure 2), suggestive of focal cerebral dysfunction predominantly in the right posterior quadrant. These EEG findings, along with the severe psychiatric symptoms, resolved after PER discontinuation (Figure 3), further supporting a causal relationship.

### Differential diagnosis consideration

Forced normalization or alternative psychosis, a phenomenon where psychiatric symptoms worsen with improvement in seizure control or normalization of the EEG,^16^ is one possibility. However, in our case, there was no clear evidence of significant seizure improvement coinciding with the exacerbation of psychiatric symptoms. Seizure frequency was consistent on a monthly basis before the initiation of PER, before admission, and after PER discontinuation. Although seizure frequency at admission had increased due to temporary discontinuation of medication secondary to persecutory delusions, it decreased concurrently with the improvement of psychiatric symptoms upon resumption of antiseizure medications. Furthermore, the EEG during the psychotic episode showed pathological LRDA, and epileptiform activity was consistently observed, indicating it was not a “normalized” EEG. Therefore, we assessed that this was not classical forced normalization.

The onset of psychiatric symptoms 14 months after reaching the final dose of PER is somewhat later than the typically reported timeframe (usually within 4 months), but delayed reactions can occur.^17^, ^18^ The rapid disappearance of both LRDA and severe psychiatric symptoms within days to weeks after PER withdrawal strongly supports the role of PER.

## CONCLUSION

We encountered a case of a PER‐induced neuropsychiatric symptom exacerbation that manifested concurrently with the appearance of LRDA. The LRDA observed in this instance bore similarities with the TIRDA noted in the interictal phase of temporal lobe epilepsy. However, the LRDA was distinctively marked by concomitant psychiatric manifestations and consistent temporal regularity. It is plausible that the LRDA in this case reflects cerebral dysfunction attributable to PER, potentially conjoined with the neuropsychiatric manifestations.

## AUTHOR CONTRIBUTIONS

The author, serving as the attending physician, was involved in clinical assessment, management, data collection, analysis, and manuscript preparation for this case report.

## CONFLICT OF INTEREST STATEMENT

The authors declare no conflicts of interest.

## ETHICS APPROVAL STATEMENT

N/A.

### PATIENT CONSENT STATEMENT

Written informed consent was obtained from the patient for publication of this case report.

### CLINICAL TRIAL REGISTRATION

N/A.

## Data Availability

Data supporting the findings of this case report are available from the corresponding author upon reasonable request.

## References

[1] Goji H , Kanemoto K . The effect of perampanel on aggression and depression in patients with epilepsy: a short‐term prospective study. Seizure. 2019 Apr;67:1–4. 30826629 10.1016/j.seizure.2019.02.009

[2] Hansen CC , Ljung H , Brodtkorb E , Reimers A . Mechanisms underlying aggressive behavior induced by antiepileptic drugs: focus on topiramate, levetiracetam, and perampanel. Behav Neurol. 2018 Nov 15;2018:2064027. 30581496 10.1155/2018/2064027 PMC6276511

[3] von Wrede R , Meschede C , Brand F , Helmstaedter C . Levetiracetam, perampanel, and the issue of aggression: a self‐report study. Epilepsy Behav. 2021 Apr;117:107806. 33621813 10.1016/j.yebeh.2021.107806

[4] Steinhoff BJ , Klein P , Klitgaard H , Laloyaux C , Moseley BD , Ricchetti‐Masterson K , et al. Behavioral adverse events with brivaracetam, levetiracetam, perampanel, and topiramate: a systematic review. Epilepsy Behav. 2021 May;118:107939. 33839453 10.1016/j.yebeh.2021.107939

[5] Kawai KKYG . A study of epilepsy patients with intellectual disability with psychiatric symptoms after treatment with perampanel. Clin Psychiatry. 2018;60(12):1393–401.

[6] Andres E , Kerling F , Hamer H , Kasper B , Winterholler M . Behavioural changes in patients with intellectual disability treated with perampanel. Acta Neurol Scand. 2017 Dec;136(6):645–53. 28568478 10.1111/ane.12781

[7] Stavropoulos I , Louden W , Queally C , Adcock J , Tristram M , Neale M , et al. Perampanel for the treatment of epilepsy; longitudinal actuarial analysis and dose responses based on monthly outcomes. Seizure. 2019 Jul;69:125–32. 31026743 10.1016/j.seizure.2019.04.013

[8] Allard J , Henley W , Snoeijen‐Schouwenaars F , Ool J , Tan I , Jurgen Schelhaas H , et al. European perspective of perampanel response in people with Intellectual Disability. Acta Neurol Scand. 2020 Sep;142(3):255–9. 32383205 10.1111/ane.13261

[9] Juhl S , Rubboli G . Add‐on perampanel and aggressive behaviour in severe drug‐resistant focal epilepsies. Funct Neurol. 2017 Oct/Dec;32(4):215–20. 29336297 PMC5762107

[10] Bragatti JA . Forced normalization revisited: new concepts about a paradoxical phenomenon. Front Integr Neurosci. 2021 Aug 27;15:736248. 34512281 10.3389/fnint.2021.736248 PMC8429494

[11] Ahn S‐J , Kim T‐J , Cha KS , Jun J‐S , Byun J‐I , Shin Y‐W , et al. Effects of perampanel on cognition and quantitative electroencephalography in patients with epilepsy. Epilepsy Behav. 2021 Feb;115:107514. 33328106 10.1016/j.yebeh.2020.107514

[12] Liguori C , Spanetta M , Izzi F , Russo A , Guerra A , Mercuri NB , et al. Perampanel increases cortical EEG fast activity in child and adult patients affected by epilepsy: a quantitative EEG study. Clin EEG Neurosci. 2021 Sep;52(5):360–70. 32762352 10.1177/1550059420947936

[13] Husari KS , Ritzl EK . Lateralized relta activity in patients with infectious or autoimmune insular lesions. Clin EEG Neurosci. 2021 Jan;52(1):61–5. 33334178 10.1177/1550059420966156

[14] Husari KS , Johnson EL , Ritzl EK . Acute and long‐term outcomes of lateralized rhythmic delta activity (LRDA) versus lateralized periodic discharges (LPDs) in critically ill patients. Neurocrit Care. 2021 Feb;34(1):201–8. 32556854 10.1007/s12028-020-01017-y

[15] De Stefano P , Vulliémoz S , Seeck M , Mégevand P . Lateralized rhythmic delta activity synchronous with hippocampal epileptiform discharges on intracranial EEG. Eur Neurol. 2020 Apr 28;83(2):225–7. 32344396 10.1159/000507394

[16] Yan Y , Wu J‐H , Peng X‐Y , Wang X‐F . Effects of antiseizure medications on alternative psychosis and strategies for their application. World J Psychiatry. 2022 Apr 19;12(4):580–7. 35582339 10.5498/wjp.v12.i4.580 PMC9048452

[17] Schulze‐Bonhage A , Hintz M . Perampanel in the management of partial‐onset seizures: a review of safety, efficacy, and patient acceptability. Patient Prefer Adherence. 2015 Aug 11;9:1143. 26316718 10.2147/PPA.S63951 PMC4542413

[18] Ramana R , Silverstone PH , Lishman WA . The toxic effects of anticonvulsant drugs in long‐term treatment of epilepsy. J Neurol Neurosurg Psychiatry. 1989 Sep 1;52(9):1116. 2795089 10.1136/jnnp.52.9.1116 PMC1031756

